# Index-based tools for livelihood security and resilience assessment (LiSeRA) in lower Mekong Basin

**DOI:** 10.1016/j.mex.2023.102301

**Published:** 2023-07-29

**Authors:** Indrajit Pal, Ayush Baskota, Ganesh Dhungana, Parmeshwar Udmale, Mayuri Ashokrao Gadhawe, Puvadol Doydee, Tanh T.N. Nguyen, Seak Sophat, Sreejita Banerjee

**Affiliations:** a Asian Institute of Technology, Pathum Thani 12120, Thailand; b Indian Institute of Technology Bombay, Mumbai 400076, India; c Kasetsart University Chalermphrakiat Sakon Nakhon Province Campus, Sakon Nakhon 47000, Thailand; d An Giang University (AGU), Vietnam National University-Ho Chi Minh City (VNU-HCM), Ho Chi Minh City, Viet Nam; e Royal University of Phnom Penh, Phnom Penh, Cambodia

**Keywords:** Resilience, AHP, Index, Climate hazard, Livelihood security, Mekong River, Disaster risk, Index Based Tools for Livelihood Security and Resilience Assessment

## Abstract

This paper presents a framework and toolkit for assessment of multi-hazard livelihood security and resilience in the Lower Mekong Basin (LMB) communities. The LMB is a subsidiary region of the Mekong region in South East Asia, and is frequently exposed to hydrometeorological hazards and anthropogenic stressors that expose and directly affect the livelihoods of more than sixty-five million people living in the region. The main purpose of the study is to support decision-making and risk management planning through integration of the concepts of livelihood security and resilience into a holistic framework, and subsequently developing an index-based toolkit for conducting assessments. Firstly, dimensions, sub-dimensions and indicators for measurement of livelihood security and resilience in the LMB were identified through comprehensive literature review and expert consultation. Then, several local workshops were conducted with various stakeholders (researchers, government officials, community people) in the LMB region to validate the indicators and generate weightages. The indicators were then arranged in a matriculated form, and the weightages were used to generate the algorithm for computing the quantitative outputs of livelihood security and resilience in study area. An Excel toolkit and a ‘R’ programming package were developed using the algorithm for visualization of the assessment outcomes. The proposed framework and toolkit are expected to assist researchers, government officials and development professionals in generating robust resilience assessment indices for risk informed decision-making and planning.

Brief outline of the method

• Livelihood security and resilience concepts were integrated to generate a holistic assessment framework and an indicator library.
• Weightages for indicators were generated using the Analytical Hierarchical Process (AHP) through consultation with relevant stakeholders.
• The indicator library was developed into an algorithm-based Excel and ‘R’ programming toolkit that provides quantitative assessment outputs.

## Graphical abstract

**Figure:**
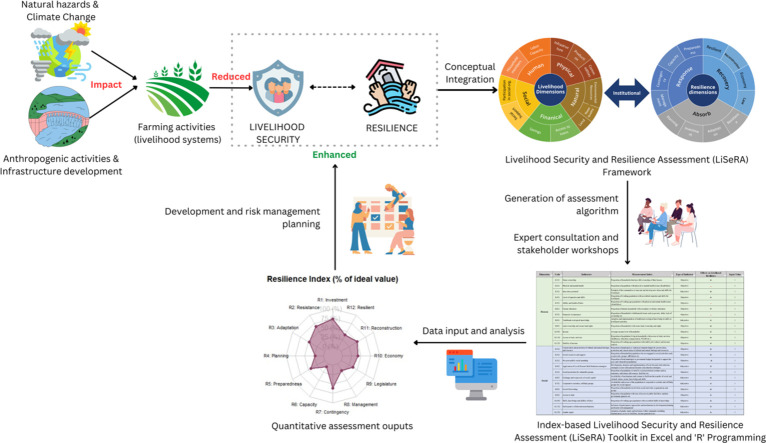

Specifications table

**Table:** 

Subject area:	Environmental Science
More specific subject area:	Risk Assessment
Name of your method:	Index Based Tools for Livelihood Security and Resilience Assessment
Name and reference of original method:	Pal, I.; Dhungana, G.; Baskota, A.; Udmale, P.; Gadhawe, M.A.; Doydee, P.; Nguyen, T.T.N.; Sophat, S. Multi-Hazard Livelihood Security and Resilience of Lower Mekong Basin Communities. **Sustainability** 2023, **15**, 8469. 10.3390/su15118469
Resource availability:	Not applicable

## Introduction

The Lower Mekong Basin (LMB) is a subsidiary region of the Mekong River, with approximately sixty-five million people directly dependent on the river for livelihood and economic activities [1]. However, communities in this region are increasingly exposed to multiple hazards that have significant direct and indirect impacts on their livelihoods [[2], [3], [4], [5]]. In the absence of robust assessment mechanisms, government agencies, development partners and local communities have not been able to design, plan and implement effective actions and strategies for livelihood security and resilience [[6], [7]]. Thus, the Livelihood Security and Resilience Assessment (LiSeRA) toolkit has been developed to enhance the quality of livelihood resilience assessment in the LMB communities. The toolkit has been developed through a comprehensive review of literature, and inputs from stakeholders including experts, government officials, and local community members who are directly involved in planning and implementing risk reduction strategies and actions [8]. The toolkit is intended to understand the linkages between livelihood and resilience and conduct quantitative assessments to generate livelihood and resilience indices for different levels (communities, local governments, and state governments).

In the first phase, a comprehensive literature review was done to develop the indicator library for the LiSeRA tool to measure the multi-hazards perspectives of the selected communities in LMB countries (Thailand, Vietnam, and Cambodia). Following the development of the indicator library, capacity-building training workshops were conducted in the project countries for the stakeholder perspectives to define weightage and ranking for the indicators through Analytical Hierarchical Process (AHP). Finally, the resilience indices have been developed through the integration of the indicators based on the LiSeRA framework specified dimensions and sub-dimensions.

## Livelihood security and resilience assessment (LiSeRA) framework

The LiSeRA framework consists of two major segments, livelihood dimension and resilience dimension, which are integrated within the governance parameters, well defined by the various legislations, guidelines, laws etc. Livelihood dimensions include five capitals derived from the theoretical principles of sustainable livelihood capitals; Human, Physical, Natural, Financial and Social [9]. These capitals are determined by a specific vulnerability context; the insecurity of one's livelihood activities and well-being in the face of ecological and environmental changes in the LMB communities. Furthermore, each livelihood capital has been divided into sub-dimensions. Human capital is measured in terms of household members and labor capacity, especially in the face of shortages of labor in the LMB due to migration and changes in trade [[10], [11]]. Similarly, the physical capacity of LMB communities is measured in terms of the status of infrastructure development and health, measures implemented to increase food production and storage, and availability, accessibility, and quality of commodities and basic services [12]. The natural capitals of the communities include the quality of ecosystem services, such as land and water, and the stock of bio-diversity and environmental resources within the community, which form essential components for the LMB communities considering their significant reliance on their environment for livelihood activities [13]. Household savings and access to loans and financial support make up the financial capital, providing reserves and buffers in preparation for potential impacts of hazards and stressors [11]. Finally, the social capital for the community includes formation, participation, and active engagement in community-based organizations, self-help groups, networks, and social support [13].

On the other hand, community-based vulnerability principles provided the foundations for resilience dimensions, further categorized into three sub-dimensions; Absorb, Response, and Recovery. The LMB communities’ capacity to absorb the impacts and stressors primarily relies on their planning and risk reduction strategies, such as flood, and water management infrastructures, advisories and early warning systems, and innovative practices, tools, and technologies in agriculture [5]. Similarly, the response capacity of communities reflects their ability to manage the impacts of threats and stressors and rely on the capability of the community to react to the changes within their environment [12]. Social bonds and support generated through community partnership and engagement programs, collaboration with government, organizations, and institutions, availability of contingent resources, and knowledge and capacity are key response capacities of the communities [14]. Finally, the recovery capacity of the LMB communities is measured by their ability to withstand and recover from the impacts of hazards, measured in terms of their institutional functions and capacities, policies, strategies, and technical and financial support for sustainable and disaster-resilient housing and settlements.

The LiSeRA toolkit library has been developed with 58 indicators for resilience and 59 indicators for livelihood security. The resilience indicators are categorized into three dimensions; Absorb, Manage/Respond and Recover, each with four sub-dimensions. Similarly, livelihood security indicators are categorized into five dimensions; human, physical, natural, social, and financial.

## Methodology

**Fig. 1:**
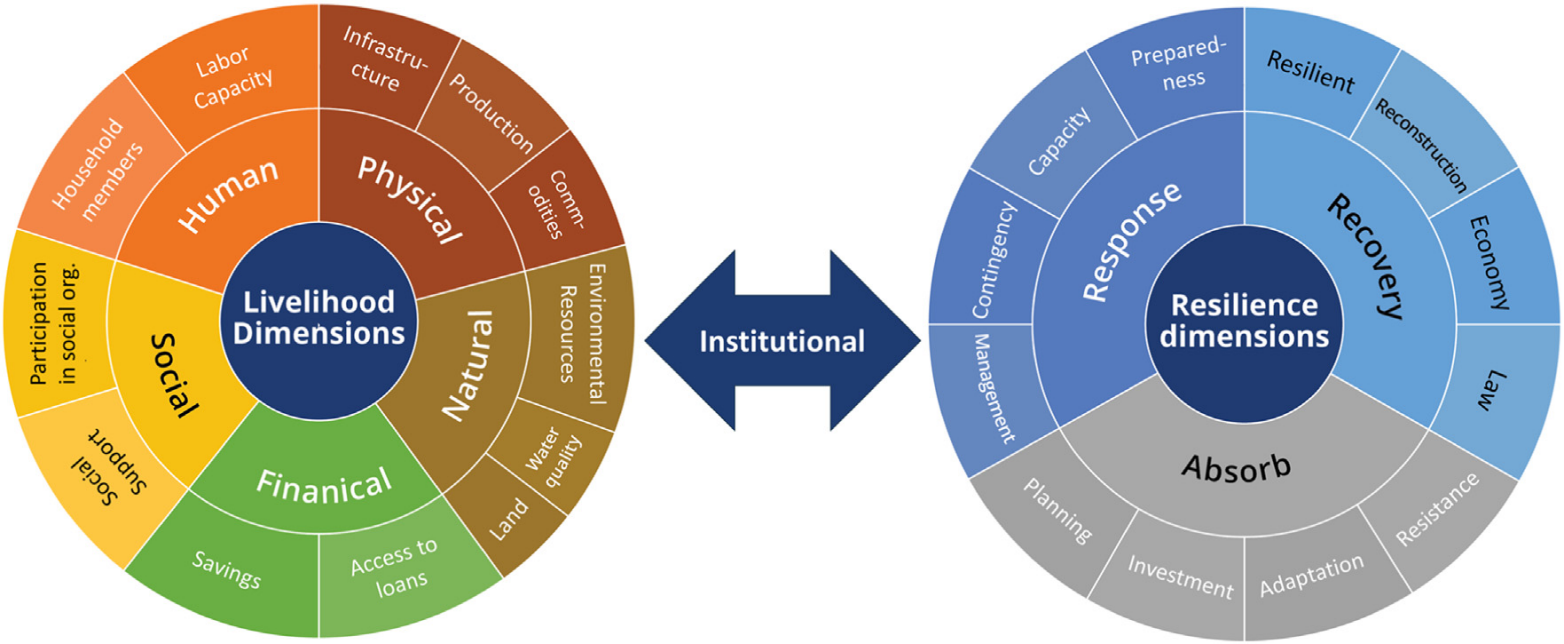
The Livelihood Security and Resilience Assessment (LiSeRA) framework depicts the dimensions and sub-dimensions [8].

**Fig. 2:**
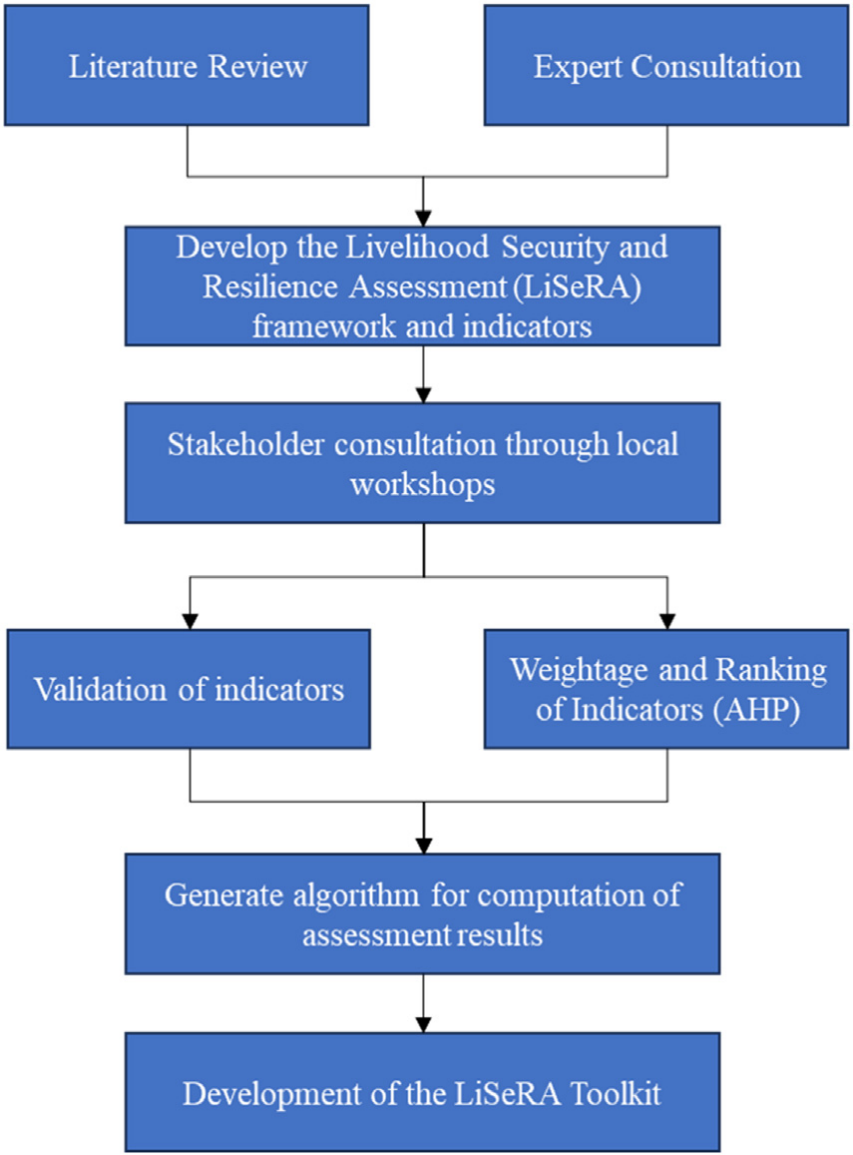
The methodological approach used in developing the LiSeRA toolkit.

The flowchart in Fig. 2 shows the methodological approach used in developing the LiSeRA Toolkit. The process was initiated through a comprehensive review of existing literature on the concepts of livelihood security and resilience in river basin communities. Several studies, reports, frameworks and policies were reviewed to understand the linkages between livelihood security and resilience in the LMB communities. These concepts were further synthesized through consultation with experts in the region, and an integrated framework (shown in Fig. 1) was developed along with identification of dimensions, sub-dimensions, indicators and the measurement parameters.

In the next stage, several local workshops were conducted with stakeholders including community people, government officials and researchers in the LMB region to discuss the assessment framework and the identified indicators. These discussions were used to validate the indicators and parameters. Additionally, the stakeholders also conducted pair-wise comparison among the indicators, which was later used to generate their comparative weightages through the Analytical Hierarchical Process (AHP). After the computation of indicator weightage, the algorithm for calculation of livelihood security and resilience indices using the indicators was developed. A six point scale (0-lowest, 5-highest) was developed to normalize the values of quantitative (objective) and qualitative (subjective) data pertinent to the indicators. This algorithm was then used to develop the LiSeRA toolkit, in MS Excel and ‘R’ programming package that provides the computed assessment indices in tabular and graphical format.

## LiSeRA toolkit

### Components of the toolkit

#### Section 1: Dashboard

The LiSeRA Microsoft Excel based toolkit has a dashboard that shows the real-time update of the calculations for livelihood and resilience dimensions indices for the study area based on the inputs given in the toolkit. Two separate dashboards have been prepared.

**Fig. 3:**
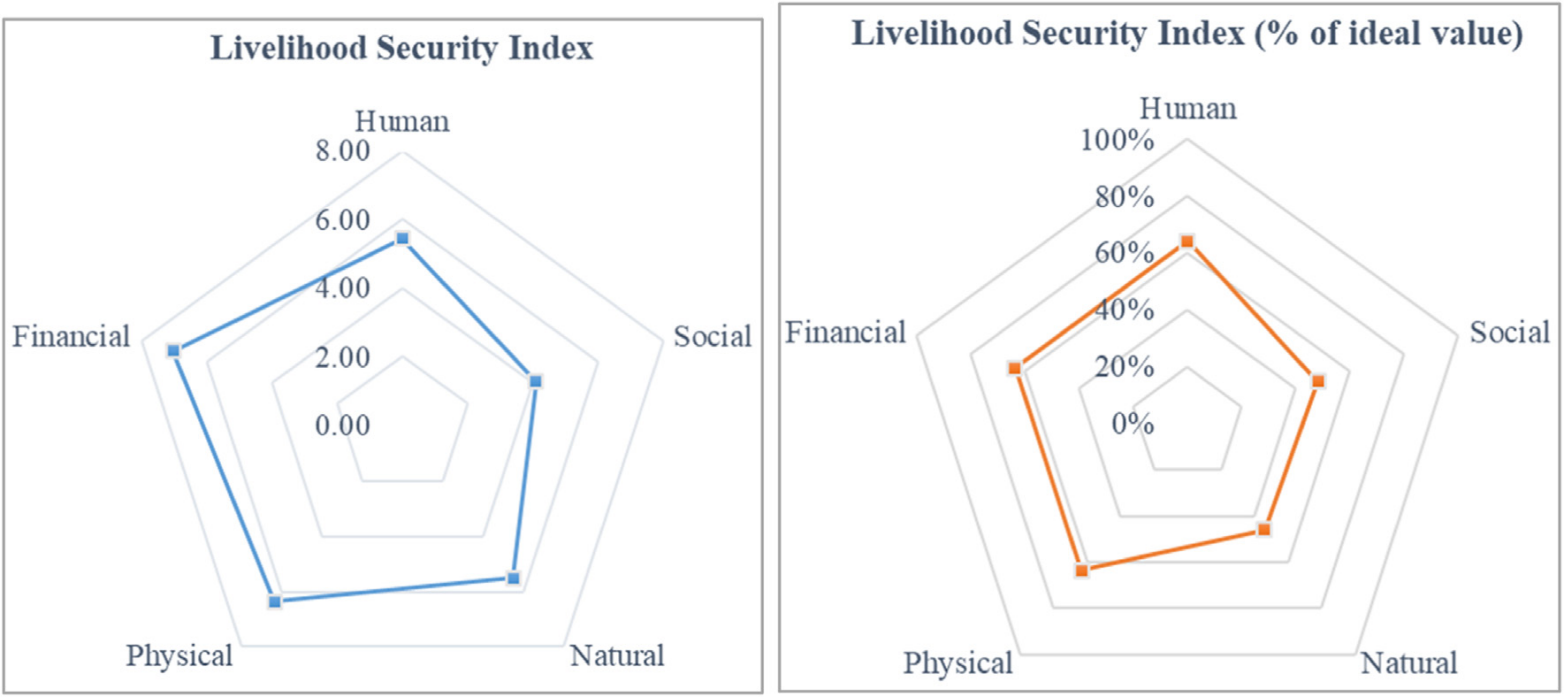
Radar plots for livelihood security indices in the LiSeRA dashboard.

Dashboard Livelihood (Table 1 and Fig. 3) shows the computed value of livelihood security dimensions (human, social, natural, physical and financial) in the form of matrix table and radar plots. Radar plots show the computed value of the different livelihood security dimensions and the percentage of ideal value.

**Table 1:** Dashboard for livelihood security dimension index.

Dimension		L1: Human		L2: Social		L3: Natural		L4:Physical		L5: Financial	
P1: Absorb	R1: Investment	L1R1	0.07	L2R1	0.17	L3R1	0.84	L4R1	0.31	L5R1	0.50
	R2: Resistance	L1R2	0.57	L2R2	0.79	L3R2	0.58	L4R2	0.00	L5R2	0.27
	R3: Adaptation	L1R3	0.22	L2R3	0.23	L3R3	0.00	L4R3	0.40	L5R3	0.64
	R4: Planning	L1R4	0.45	L2R4	0.12	L3R4	0.72	L4R4	0.27	L5R4	0.33
P2: Manage/ Respond	R5: Preparedness	L1R5	0.85	L2R5	0.00	L3R5	0.58	L4R5	1.11	L5R5	0.18
	R6: Capacity	L1R6	0.00	L2R6	0.41	L3R6	0.62	L4R6	0.56	L5R6	0.40
	R7: Contingency	L1R7	1.29	L2R7	0.30	L3R7	0.29	L4R7	0.10	L5R7	0.68
	R8: Management	L1R8	0.10	L2R8	0.38	L3R8	0.77	L4R8	0.29	L5R8	1.01
P3: Recover	R9: Legislature	L1R9	0.32	L2R9	0.28	L3R9	0.00	L4R9	1.43	L5R9	0.48
	R10: Economy	L1R10	0.36	L2R10	0.37	L3R10	0.36	L4R10	0.23	L5R10	1.90
	R11: Reconstruction	L1R11	0.47	L2R11	0.71	L3R11	0.48	L4R11	0.35	L5R11	0.38
	R12: Resilient	L1R12	0.75	L2R12	0.35	L3R12	0.29	L4R12	1.31	L5R12	0.26
SUM		5.5		4.1		5.5		6.4		7.0	

* Data for representation purposes only.

**Fig. 4:**
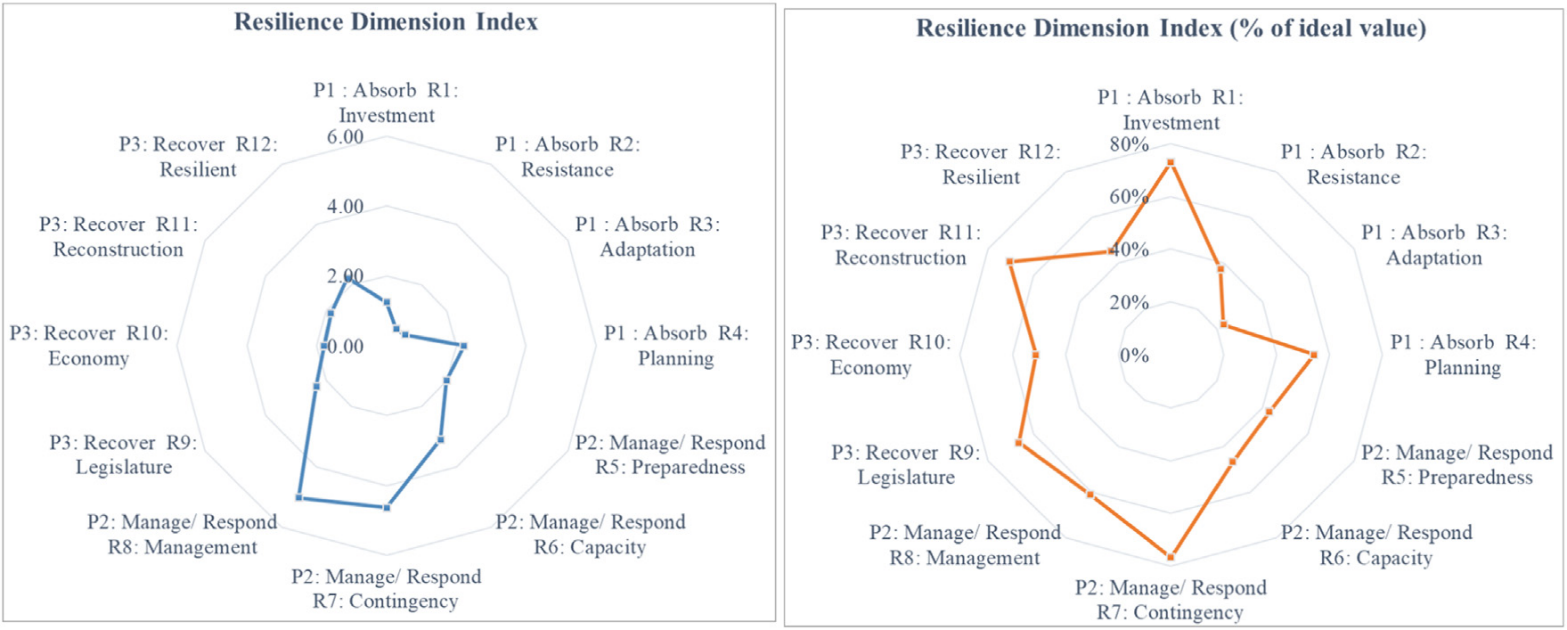
Radar plots for resilience dimension indices in the LiSeRA dashboard.
• Dimension and sub-dimension values: The matrix displays the values of each indicator computed through the calculation of indicator weightage and input value.
• Sum: The total sum for dimension (or sub dimension) for livelihood and resilience is shown.
• % of ideal value: The percentage of sum of livelihood or resilience dimensions and sub-dimensions with respect to the ideal value (generated through ideal inputs)

Dashboard Resilience (Table 2 and Fig. 4) shows the computed values of resilience sub-dimensions (investment, resistance, adaptation, planning, preparedness, capacity, contingency, management, legislature, economy, reconstruction & resilience). The computed resilience indices are displayed in the form of a matrix table (Table 2) as well as radar plots showing the computed values and percentage of ideal value (Fig. 4).

**Table 2:** Dashboard for resilience dimension index.

Dimension		L1: Human		L2: Social		L3: Natural		L4: Physical		L5: Financial		SUM
P1: Absorb	R1: Investment	R1L1	0.34	R1L2	0.17	R1L3	0.31	R1L4	0.43	R1L5	0.00	1.24
	R2: Resistance	R2L1	0.17	R2L2	0.06	R2L3	0.10	R2L4	0.07	R2L5	0.17	0.56
	R3: Adaptation	R3L1	0.13	R3L2	0.22	R3L3	0.11	R3L4	0.00	R3L5	0.16	0.62
	R4: Planning	R4L1	0.52	R4L2	0.34	R4L3	0.54	R4L4	0.36	R4L5	0.46	2.22
P2: Manage/ Respond	R5: Preparedness	R5L1	0.36	R5L2	0.37	R5L3	0.52	R5L4	0.00	R5L5	0.74	1.99
	R6: Capacity	R6L1	0.42	R6L2	0.24	R6L3	0.88	R6L4	0.37	R6L5	1.19	3.10
	R7: Contingency	R7L1	0.92	R7L2	1.14	R7L3	1.59	R7L4	0.00	R7L5	0.99	4.64
	R8: Management	R8L1	1.88	R8L2	1.81	R8L3	0.00	R8L4	0.00	R8L5	1.34	5.03
P3: Recover	R9: Legislature	R9L1	0.38	R9L2	0.26	R9L3	0.77	R9L4	0.00	R9L5	0.90	2.32
	R10: Economy	R10L1	0.00	R10L2	1.17	R10L3	0.00	R10L4	0.61	R10L5	0.00	1.78
	R11: Reconstruction	R11L1	0.00	R11L2	0.00	R11L3	1.33	R11L4	0.51	R11L5	0.00	1.84
	R12: Resilient	R12L1	0.00	R12L2	0.43	R12L3	0.37	R12L4	0.77	R12L5	0.65	2.23

* Data for representation purposes only.

#### Section 2: Indicator library

The indicator library provides a brief overview of the dimensions, sub-dimensions, and indicators in the livelihood and resilience indices. Along with this, it also displays the weightages assigned to each of the dimensions, sub-dimensions and indicators and allows for modification (if required) during the assessment process.

• Dimension/sub dimension weightage (D Wt./SD Wt.): The weightage for the dimension and/or sub-dimension within the livelihood or resilience index.
• Code: The unique code representation for the indicator.
• Indicator: The indicators within each dimension and sub-dimension
• Indicator description (measurement): A detailed description of the indicator including the most suitable index of measurement (qualitative or quantitative).
• Indicator Wt.: The weightage assigned to the indicators.
• Adjusted Indicator Wt**:** The adjusted value of indicator weightage is computed automatically by multiplying the dimension, sub-dimension weightages and the indicator weightages.
• References: Sources of journal articles, publications and documents for the respective indicators.

#### Section 3: Input values

This sheet will be used to input the values for the individual indicators based on the subjective or objective assessment of the study area by the user. Separate sheets have been designed for livelihood and resilience indices. The instructions for placing the input values are shown in the top row of the sheet and described in the following sections. In addition to the fields already described earlier in the indicator library, the input values have the following fields,

• Type of indicator: Displays the type of indicator and measurement index (subjective or objective)
• Effect on livelihood/resilience: The effect of the indicator on the livelihood or resilience indices (positive indicators will increase livelihood or resilience, and negative will decrease)
• Input value: The input value for the particular indicator computed based on the subjective or objective assessment of the study area by the researcher. The input value ranges from 0 (Absent/Very Low) to 5 (Very High).
• Indicator Wts: The adjusted weightages for each indicator (refered to from the indicator library sheet).
• Value: The total value of the indicator in the livelihood or resilience index is computed by multiplying the input value with the indicator weightage.

### Using the LiSeRA excel toolkit for assessment

***Step 1: Select and input the dimension, sub-dimension, and indicator weightage for the assessment*.**

• The weightages for dimensions, sub-dimensions and indicators must be input in the Indicator Library sheets (Tables 3 & 4)

**Table 3:** Excerpt of indicator library for livelihood security dimensions.

Instructions	Enter the weightage of Livelihood Dimensions in this Column				Enter the weightage of each indicator in this column	The adjusted indicator weightage will be calculated automatically from the entered dimension and indicator weightage	
Dimension	Dim Wt.	Code	Indicators	Indicator Description (Measurement)	Ind Wt.	Adjusted Indicator Wt = Gobal Wt * Indicator Wt	Refs.
Human	0.17	L1R1	Home ownership	Proportion of households that have full ownership of their houses	0.04	0.007	[11]
		L1R2	Physical and mental health	Proportion of population with physical or mental health issues (disabilities)	0.11	0.019	[11]
		L1R3	Innovation potential	Potential of the communities to innovate and develop new ideas and skills for livelihood	0.03	0.004	(Oxfam, 2013)
		L1R4	Level of expertise and skills	Proportion of working population with accredited expertise and skills for livelihood	0.07	0.011	[11]
		L1R5	Ability and health of labor	Proportion of working age population with physical and mental health issues (disabilities)	0.25	0.043	(Scoones, 1998)
		L1R6	Farmer education	Proportion of farmer households with secondary or tertiary education	0.05	0.008	[13]
		L1R7	Financial circumstance	Proportion of households with financial issues such as poverty, debts, lack of ownership etc.	0.15	0.026	[11]
		L1R8	Traditional ecological knowledge	Adoption and implementation of traditional ecological knowledge & skills in livelihood activities	0.03	0.005	[13]
		L1R9	Land ownership and secure land rights	Proportion of households with secure land ownership and rights	0.05	0.008	(SDG, 2017)
		L1R10	Income	Average income level of households	0.07	0.012	[10]
		L1R11	Access to basic services	Proportion of population living in households with access to basic services (healthcare, education, transportation, WASH etc.)	0.07	0.012	(SDG, 2017)
		L1R12	Stability of income	Proportion of working-age population with stable jobs (salary) and income sources	0.09	0.015	[11]

* Full table available in the supplementary material.

**Table 4:** Excerpt of indicator library for resilience dimensions.

INSTRUCTIONS	Enter the Global Weightage of Resilience Dimensions here		Enter the weightages of the Sub-dimensions in each dimension here				Enter the weightages of indicators within each of the sub-dimension here	The adjusted indicator weightage will be calculated automatically from the entered dimension and indicator weightage	
Dimension	Dim Wt.	Sub -Dimension	Sub Dim Wt.	Code	Indicators	Indicator Description (Measurement)	Ind. Wt	Adjusted Indicator Wt = D Wt. * SD Wt.*Ind Wt.	Refs.
Absorb	0.2	Investment	0.17	R1L1	Comprehensive partnership with external agencies on DRR	Actions undertaken for enhancing partnerships with external agencies for disaster risk management	0.20	0.007	[12]
				R1L2	Investment in management and conservation of natural resources.	Proportion of investment from community and local governments to sustain natural resources	0.25	0.009	(Courtney et al., 2008)
				R1L3	Developers and communities incorporate risk reduction into the location and design of structures.	Risk reduction measures incorporated into the location and design of structures	0.30	0.010	(Courtney et al., 2008)
				R1L4	Ownership of farming equipment (own, rent, borrow pieces of equipment)	Proportion of agricultural households who have ownership of farming equipment	0.25	0.009	(Quandt, 2018)
		Resistance	0.15	R2L1	Social support and network systems on DRR activities	Presence of social support and systems for implementation of disaster risk reduction activities to reduce hazard impacts	0.14	0.004	[12]
				R2L2	Protection and enhancement of ecosystem and bio-diversity	Sensitive habitats, ecosystems, and natural features protect and maintained to reduce risk from hazards	0.19	0.006	(Courtney et al., 2008)
				R2L3	Irrigation for productive agriculture	Proportion of agricultural land area equipped with irrigation facilities for productive agriculture	0.16	0.005	(Quandt, 2018)
				R2L4	Access to public services	Proportion of population with easy access to public facilities (schools, hospitals)	0.22	0.007	(Quandt, 2018)
				R2L5	Income stability	Proportion of population with stable income (salaried jobs)	0.29	0.009	(Quandt, 2018)
		Adaptation	0.27	R3L1	Education Level	Proportion of population with secondary and tertiary level of education	0.24	0.013	[10]
				R3L2	Social protection and safety of vulnerable groups	Proportion of vulnerable population covered by social protection systems (insurances, benefits, support system etc.)	0.20	0.011	[12]
				R3L3	Indigenous knowledge and technologies	Application of indigeneous knowledge and technologies in farming and other livelihood activities	0.21	0.011	[12]
				R3L4	Availability of robust road network	Proportion of 'all-season' roads in comparison to total road network within community/local government	0.20	0.011	(Quandt, 2018)
				R3L5	Remittance	Proportion of remittance and income from external sources as a source of income for the community	0.15	0.008	(Quandt, 2018)
		Planning	0.41	R4L1	Education Level	Proportion of population with secondary and tertiary level of education	0.21	0.017	[14]
				R4L2	Community-Based Planning	Presence of community based approaches in planning of DRM and development activities	0.21	0.017	(US Department of Homeland Security, 2016)
				R4L3	Protected areas and ecosystems	Proportion of important sites for terrestrial and freshwater biodiversity that are covered by protected areas, by ecosystem type	0.22	0.018	(SDG, 2017)
				R4L4	Investment in early warning and evacuation system	Financial resources available to maintain and improve warning and evacuation systems	0.22	0.018	(Courtney et al., 2008)
				R4L5	Access to a bank account	Proportion of population with ownership of accounts in financial institutions (banks)	0.14	0.011	(Quandt, 2018b)

* Full table available in the supplementary material.
• The general instructions for inserting the values in the sheet are placed in the first row.
• The current weightages of the indicators in the LiSeRA toolkit have been generated from pairwise comparison and AHP based on the opinions of experts and stakeholders in the LMB communities.
• User may choose to continue your assessment with the same weightages, or generate and enter new weightages based on your own indicator weightage assessment process.
• The weightages of the respective dimensions and indicators must be placed in the ‘Indicator Library_Livelihood’ and ‘Indicator Library_Resilience’ sheets.
• In the ‘Indicator Library Livelihood’ sheet, the global weightage for five Livelihood dimensions (human, social, natural, physical, and financial) is entered in the column marked ‘Dim Wt.’. Similarly, indicator weightages is entered in the column marked ‘Ind. Wt.’. It must be ensured that the sum of weightages for five dimensions and twelve indicators in each dimension equals to 1 (One).
• The similar process must be done to enter the weightages of the dimension and indicators in the resilience dimensions, in the “Indicator Library_Resilience” sheet.

***Step 2: Input values for livelihood and resilience dimension indicators***

• The values for indicators within the livelihood and resilience dimension must be placed in the ‘Input Values_Livelihood’ and ‘Input Values_Resilience’ sheets respectively (Tables 5 & 6)

**Table 5:** Excerpt of sheet for input values for livelihood security dimension indicators in LiSeRA Toolkit.

INSTRUCTIONS			Check the measurement index (description of indicator including the metric of measurement)	Check the type of indicator (subjectiive or objective)	Check the effect of the indicator on the Livelihood Resilience (positive or negative)	Input the assessment value for the indicator	The indicator weightage is automatically copied from previous sheet	Adjusted value of the indicator will be automatically calculated
A	B	C	D	E	F	G	I	H
Dimension	Code	Indicators	Indicator Description (Measurement)	Type of Indicator	Effects on Livelihood Resilience	Input Value	Indicator Wts	Value = Input * Indicator Weightage * 10
Human	L1R1	Home ownership	Proportion of households that have full ownership of their houses	Objective	Positive	1	0.007	0.074
	L1R2	Physical and mental health	Proportion of population with physical or mental health issues (disabilities)	Objective	Negative	2	0.019	0.568
	L1R3	Innovation potential	Potential of the communities to innovate and develop new ideas and skills for livelihood	Subjective	Positive	5	0.004	0.218
	L1R4	Level of expertise and skills	Proportion of working population with accredited expertise and skills for livelihood	Objective	Positive	4	0.011	0.447
	L1R5	Ability and health of labor	Proportion of working age population with physical and mental health issues (disabilities)	Objective	Negative	3	0.043	0.851
	L1R6	Farmer education	Proportion of farmer households with secondary or tertiary education	Objective	Positive	0	0.008	0.000
	L1R7	Financial circumstance	Proportion of households with financial issues such as poverty, debts, lack of ownership etc.	Objective	Negative	0	0.026	1.294
	L1R8	Traditional ecological knowledge	Adoption and implementation of traditional ecological knowledge & skills in livelihood activities	Subjective	Positive	2	0.005	0.102
	L1R9	Land ownership and secure land rights	Proportion of households with secure land ownership and rights	Objective	Positive	4	0.008	0.319
	L1R10	Income	Average income level of households	Objective	Positive	3	0.012	0.359
	L1R11	Access to basic services	Proportion of population living in households with access to basic services (healthcare, education, transportation, WASH etc.)	Objective	Positive	4	0.012	0.470
	L1R12	Stability of income	Proportion of working-age population with stable jobs (salary) and income sources	Objective	Positive	5	0.015	0.748

* Full table available in the supplementary material.

**Table 6:** Excerpt of sheet for input values for resilience dimension indicators in LiSeRA Toolkit.

INSTRUCTIONS	Enter the Global Weightage of Resilience Dimensions here		Enter the weightages of the Sub-dimensions in each dimension here				Enter the weightages of indicators within each of the sub-dimension here	The adjusted indicator weightage will be calculated automatically from the entered dimension and indicator weightage	
Dimension	Dim Wt.	Sub -Dimension	Sub Dim Wt.	Code	Indicators	Indicator Description (Measurement)	Ind. Wt	Adjusted Indicator Wt = D Wt. * SD Wt.*Ind Wt.	Refs.
Absorb	0.2	Investment	0.17	R1L1	Comprehensive partnership with external agencies on DRR	Actions undertaken for enhancing partnerships with external agencies for disaster risk management	0.20	0.007	[12]
				R1L2	Investment in management and conservation of natural resources.	Proportion of investment from community and local governments to sustain natural resources	0.25	0.009	(Courtney et al., 2008)
				R1L3	Developers and communities incorporate risk reduction into the location and design of structures.	Risk reduction measures incorporated into the location and design of structures	0.30	0.010	(Courtney et al., 2008)
				R1L4	Ownership of farming equipment (own, rent, borrow pieces of equipment)	Proportion of agricultural households who have ownership of farming equipment	0.25	0.009	(Quandt, 2018)
		Resistance	0.15	R2L1	Social support and network systems on DRR activities	Presence of social support and systems for implementation of disaster risk reduction activities to reduce hazard impacts	0.14	0.004	[12]
				R2L2	Protection and enhancement of ecosystem and bio-diversity	Sensitive habitats, ecosystems, and natural features protect and maintained to reduce risk from hazards	0.19	0.006	(Courtney et al., 2008)
				R2L3	Irrigation for productive agriculture	Proportion of agricultural land area equipped with irrigation facilities for productive agriculture	0.16	0.005	(Quandt, 2018)
				R2L4	Access to public services	Proportion of population with easy access to public facilities (schools, hospitals)	0.22	0.007	(Quandt, 2018)
				R2L5	Income stability	Proportion of population with stable income (salaried jobs)	0.29	0.009	(Quandt, 2018)
		Adaptation	0.27	R3L1	Education Level	Proportion of population with secondary and tertiary level of education	0.24	0.013	[10]
				R3L2	Social protection and safety of vulnerable groups	Proportion of vulnerable population covered by social protection systems (insurances, benefits, support system etc.)	0.20	0.011	[12]
				R3L3	Indigenous knowledge and technologies	Application of indigeneous knowledge and technologies in farming and other livelihood activities	0.21	0.011	[12]
				R3L4	Availability of robust road network	Proportion of 'all-season' roads in comparison to total road network within community/local government	0.20	0.011	(Quandt, 2018)
				R3L5	Remittance	Proportion of remittance and income from external sources as a source of income for the community	0.15	0.008	(Quandt, 2018)
		Planning	0.41	R4L1	Education Level	Proportion of population with secondary and tertiary level of education	0.21	0.017	[14]
				R4L2	Community-Based Planning	Presence of community based approaches in planning of DRM and development activities	0.21	0.017	(US Department of Homeland Security, 2016)
				R4L3	Protected areas and ecosystems	Proportion of important sites for terrestrial and freshwater biodiversity that are covered by protected areas, by ecosystem type	0.22	0.018	(SDG, 2017)
				R4L4	Investment in early warning and evacuation system	Financial resources available to maintain and improve warning and evacuation systems	0.22	0.018	(Courtney et al., 2008)
				R4L5	Access to a bank account	Proportion of population with ownership of accounts in financial institutions (banks)	0.14	0.011	(Quandt, 2018b)

⁎ Full table available in the supplementary material.
• The general instructions for inserting the values in the sheet are placed in the first row.
• Before inserting the values for the indicators, measurement index (description of indicator including the metric of measurement), the type of indicator (subjective or objective), and the effect on livelihood or resilience (positive or negative) must be checked carefully.
• Input values must be accurately chosen based on the type of indicator and the effect of the indicator on the livelihood and resilience dimensions as follows,
  ○ For objective indicators, a normalization scale must be developed to categorize data obtained for the study area into six levels: 0 – lowest, 5 – highest.
  ○ For subjective indicators, subjective assessment must be conducted in the study area. The information collected must be categorized into six categories and respective input values as shown in the Table 7.

**Table 7:** Input values for subjective indicators based on their status in study area.

Status	Benchmark	Input Value
Absent	Absent Condition	0
Poor	1–20% fulfilled	1
Fair	21–40% Fulfilled	2
Good	41–60% Fulfilled	3
Very Good	61–80% Fulfilled	4
Excellent	80–100% fulfilled	5
• In the case of both positive and negative indicators, the input data will follow the same scaling. For both indicators, only whole numbers should be entered, without any signs (-/+). The toolkit has been calibrated to automatically assign negative values to the negative indicators.
• After determining the values for the indicators, the input values must be placed in the respective columns; Column G in ‘Input Values_Livelihood’ sheet (Table 5) and Column H in the ‘Input Values_Resilience’ sheet (Table 6).
• Once the values for the indicators are placed in the respective fields, the adjusted values are automatically calculated by multiplying the input value with the indicator weightage.

***Step 3: Check tabular and graphical outputs***

• The outputs of the livelihood security and resilience will be generated in the LiSeRA dashboard.
• The outputs will be generated in tabular (matrix) and graphical form through radar plots (see Tables 1, 2 and Figs. 2, 3)

## Conclusion

The LiSeRA toolkit has been developed through a comprehensive review of literature, and inputs from stakeholders including experts, government officials, and local community members who are directly involved in planning and implementing risk reduction strategies and actions. The toolkit is intended for researchers, development planners, and policymakers to understand the linkages between livelihood and resilience and conduct quantitative assessments to generate livelihood and resilience indices for different levels (communities, local governments, and state governments). Additionally, the toolkit also serves as a reference for researchers and practitioners in identifying critical indicators that can be used to plan and evaluate risk assessment and management actions within the LMB communities. Livelihood security encompasses the people's ability to cope with natural and anthropogenic hazards and threats such as irregular weather patterns, resource constraints, epidemics and diseases, and market fluctuations that contribute to the sustainability of their livelihoods. Future research should focus on developing well-informed resilience status maps, which include the interdependency parameters of various infrastructures, such as hydrological infrastructures that support livelihood (e.g., dams, water gates, irrigation canals, freshwater ponds, water reservoirs etc.), electricity, transit as well as interactions between other systems. Finally, the LiSeRA framework focuses on assessing livelihood security in the river basin system; however, its application can be extended to a wide range of livelihoods and other geophysical settings.

## CRediT authorship contribution statement

**Indrajit Pal:** Conceptualization, Methodology, Data curation, Formal analysis, Writing – original draft, Writing – review & editing. **Ayush Baskota:** Methodology, Formal analysis, Writing – review & editing. **Ganesh Dhungana:** Conceptualization, Methodology, Data curation, Formal analysis, Writing – original draft, Writing – review & editing. **Parmeshwar Udmale:** Conceptualization, Methodology, Data curation, Formal analysis, Writing – original draft, Writing – review & editing. **Mayuri Ashokrao Gadhawe:** Methodology, Data curation, Formal analysis, Software. **Puvadol Doydee:** Methodology, Data curation, Writing – review & editing. **Tanh T.N. Nguyen:** Methodology, Data curation, Writing – review & editing. **Seak Sophat:** Methodology, Data curation, Writing – review & editing. **Sreejita Banerjee:** Methodology, Writing – review & editing.

## Declaration of Competing Interest

The authors declare that they have no known competing financial interests or personal relationships that could have appeared to influence the work reported in this paper.

## Data Availability

Data will be made available on request.

## Appendix Supplementary materials

Image, application 1

## References

[1] Morton L.W., Olson K.R. (2018). The pulses of the Mekong River Basin: rivers and the livelihoods of farmers and fishers. J. Environ. Prot..

[2] Dang A.T.N., Kumar L., Reid M., Nguyen H. (2021). Remote sensing approach for monitoring Coastal Wetland in the Mekong Delta, Vietnam: change trends and their driving forces. Remote Sens..

[3] Dang T.D., Cochrane T.A., Arias M.E., Tri V.P.D. (2018). Future hydrological alterations in the Mekong Delta under the impact of water resources development, land subsidence and sea level rise. J. Hydrol. Reg. Stud..

[4] Hoang L.P., Biesbroek R., Tri V.P.D., Kummu M., van Vliet M.T.H., Leemans R., Kabat P., Ludwig F. (2018). Managing flood risks in the Mekong Delta: how to address emerging challenges under climate change and socioeconomic developments. AMBIO.

[5] Kura Y., Joffre O., Laplante B., Sengvilaykham B. (2017). Coping with resettlement: a livelihood adaptation analysis in the Mekong River basin. Land Use Policy.

[6] Nguyen T.P.L., Sean C. (2021). Do climate uncertainties trigger farmers’ out-migration in the Lower Mekong region?. Curr. Res. Environ. Sustain..

[7] Thilakarathne M., Sridhar V. (2017). Characterization of future drought conditions in the Lower Mekong River Basin. Weather Clim. Extrem..

[8] Pal I., Dhungana G., Baskota A., Udmale P., Gadhawe M.A., Doydee P., Nguyen T.T.N., Sophat S. (2023). Multi-hazard livelihood security and resilience of lower Mekong Basin communities. Sustainability.

[9] DFID. (1999). Sustainable livelihoods guidance sheets.

[10] Ainuddin S., Routray J.K. (2012). Earthquake hazards and community resilience in Baluchistan. Nat. Hazards.

[11] Sina D., Chang-Richards A.Y., Wilkinson S., Potangaroa R. (2019). A conceptual framework for measuring livelihood resilience: relocation experience from Aceh, Indonesia. World Dev..

[12] Orencio P.M., Fujii M. (2013). A localized disaster-resilience index to assess coastal communities based on an analytic hierarchy process (AHP). Int. J. Disaster Risk Reduct..

[13] Lecegui A., Olaizola A.M., López-i-Gelats F., Varela E. (2022). Implementing the livelihood resilience framework: an indicator-based model for assessing mountain pastoral farming systems. Agric. Syst..

[14] Ifejika Speranza C., Wiesmann U., Rist S. (2014). An indicator framework for assessing livelihood resilience in the context of social–ecological dynamics. Glob. Environ. Chang..

